# Benzodeazaoxaflavin Sirtuin Inhibitors Inhibit *Schistosoma mansoni* Sirt2 and Cause Phenotypic Changes and Lethality in Schistosomula and Adult Worm Stages

**DOI:** 10.1021/acsinfecdis.5c00515

**Published:** 2025-10-07

**Authors:** Roberto Gimmelli, Giuliana Papoff, Emanuele Fabbrizi, Michela Guida, Cristiana Lalli, Fulvio Saccoccia, Cécile Häberli, Jennifer Keiser, Daria Monaldi, Manfred Jung, Christophe Romier, Dante Rotili, Antonello Mai, Giovina Ruberti

**Affiliations:** ^ Institute of Biochemistry and Cell Biology, National Research Council (IBBC−CNR), Adriano Buzzati-Traverso Campus, Monterotondo, Rome 00015, Italy; † Department of Drug Chemistry and Technologies, 9311 Sapienza University of Rome, Rome 00185, Italy; ¥ 30247 Swiss Tropical and Public Health Institute, Allschwil 4002, Switzerland; + University of Basel, Basel 4001, Switzerland; # Institute of Pharmaceutical Sciences, Albert-Ludwigs-Universität Freiburg, Freiburg 79104, Germany; ∥ Département de Biologie Structurale Intégrative, Université de Strasbourg, CNRS, INSERM, Institut de Génétique et de Biologie Moléculaire et Cellulaire (IGBMC), Illkirch Cedex 67404, France; ϕ Department of Science, Roma Tre University, Viale Guglielmo Marconi 446, Rome 00146, Italy; Δ Biostructures and Biosystems National Institute (INBB), Via dei Carpegna 19, Rome 00165, Italy

**Keywords:** sirtuins, SmSirt2 inhibitors, *Schistosoma mansoni*, phenotypic and biochemical characterization

## Abstract

Schistosomiasis, a neglected tropical disease caused by trematodes of *Schistosoma* genus, urgently requires new treatments due to praziquantel’s limited efficacy against juvenile worms as well as the threat of drug resistance. In this study, we evaluated a series of benzodeazaoxaflavin (BDF4)-based compounds as inhibitors of the parasite’s epigenetic enzyme *Sm*Sirt2. Three compounds, **7**–**9** (MC2346, MC2141, and MC2345), showed activity against both Liberian and Puerto Rican strains of *Schistosoma mansoni*. The compounds reduced schistosomula and adult worm pair viability, pairing, and egg production, with low cytotoxicity in mammalian cells. These effects were linked to histone H3 hyperacetylation and cytochrome c-mediated apoptosis, confirming *Sm*Sirt2 as a functional target. These findings support the development of *Sm*Sirt2 inhibitors as novel antischistosomal agents with therapeutic potential for both curative and preventive applications. Further *in vivo* studies are warranted to assess their pharmacokinetic and safety profiles.

## Graphical abstract

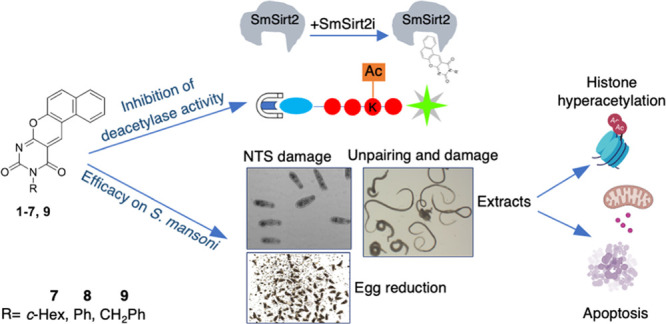

Schistosomes are the only parasitic platyhelminths that have evolved separate sexes, exhibiting a unique reproductive biology in which female sexual maturation depends on constant pairing with a male. Schistosomes cause schistosomiasis, a neglected tropical disease of global importance affecting both humans and animals. The pathology of schistosomiasis is triggered by eggs released in the bloodstream by paired females. When eggs become trapped in organs such as the liver, they cause inflammation, granulomatous reactions, and fibrosis.^1^ Continuous physical contact with a male is essential for the full development of female gonads. Notably, the female resides within a ventral groove formed by the male’s gynecophoral canal.^2,3^ Males induce mitosis and differentiation in the female reproductive organs (ovary and vitellarium),^4–6^ and pairing regulates the expression of female-specific genes involved in vitellarium function.^7,8^ Recent RNA-seq analyses of paired and unpaired adults and their gonads revealed several differentially expressed mRNAs and long noncoding RNAs,^9–11^ suggesting a reciprocal relationship that affects not only the gonads but also other signaling and biological processes in both sexes.^12^

Praziquantel (PZQ) remains the only approved drug for the treatment of schistosomiasis.^13–15^ Its administration through mass drug administration (MDA) so-called preventive chemotherapy programs in a single dose is the cornerstone of schistosomiasis control. However, the extensive and growing use of PZQ for its use in MDA at regular intervals to at-risk populations decreases drug efficacy and raises concerns about the emergence of drug resistance in schistosome populations.^16,17^ Although PZQ is easy to administer, safe, well-tolerated, and inexpensive, it shows limited activity against juvenile worms and cannot prevent reinfection in at-risk populations. Preliminary evidence of reduced susceptibility in children repeatedly exposed to MDA has been reported.^18^ Therefore, drugs capable of eliminating both juvenile and adult stages of major *Schistosoma* species (*S. mansoni*, *S. hematobium*, and *S. japonicum*) with equal efficacy are urgently needed to provide fully curative treatment.

Due to the similarities between parasites and cancer cells^19^ including metabolic adaptation,^20^ immune evasion,^21^ and cellular invasion and migration,^22^ anticancer drugs, particularly epigenetic inhibitors, are being investigated for antiparasitic activity.^23–25^

Recently, we reported the use of inhibitors targeting parasite-specific epigenetic regulators such as *Sm*HDAC8,^26–29^ *Sm*Sirt2,^30^ and *Sm*LSD1 ^31^ as antischistosomal agents. Sirtuins, class III histone deacetylases, possess a NAD^+^-dependent catalytic mechanism of action.^32^ Seven Sirtuins have been identified in *S. mansoni*, expressed throughout its lifecycle, and inhibitors of human Sirtuins have shown lethal effects on schistosomula, disrupted adult worm pairing, and caused tissue damage in reproductive organs.^33^

Specifically, salermide (Figure 1), an hSirt1/hSirt2 inhibitor previously identified by our research group for its selective proapoptotic activity in human cancer cells^34,35^ and protective effects in a nematode model of muscular dystrophy,^36^ induced DNA fragmentation in schistosomula and significantly impaired adult worm pairing and egg production, while disrupting ovary and testis morphology.^33^ Furthermore, a series of *N* ^7^-(4-phenoxybenzyl)­pyrimido­[4,5-*d*]­pyrimidine-2,4,7-triamines (e.g., compound **29** in Figure 1), identified through screening of a focused GSK Kinetobox library and subsequent fragment-based optimization, demonstrated specific *Sm*Sirt2 inhibition. These compounds reduced schistosomula viability, impaired adult pairing and egg laying, and exhibited low cytotoxicity in human cells.^30^

**Figure 1:**
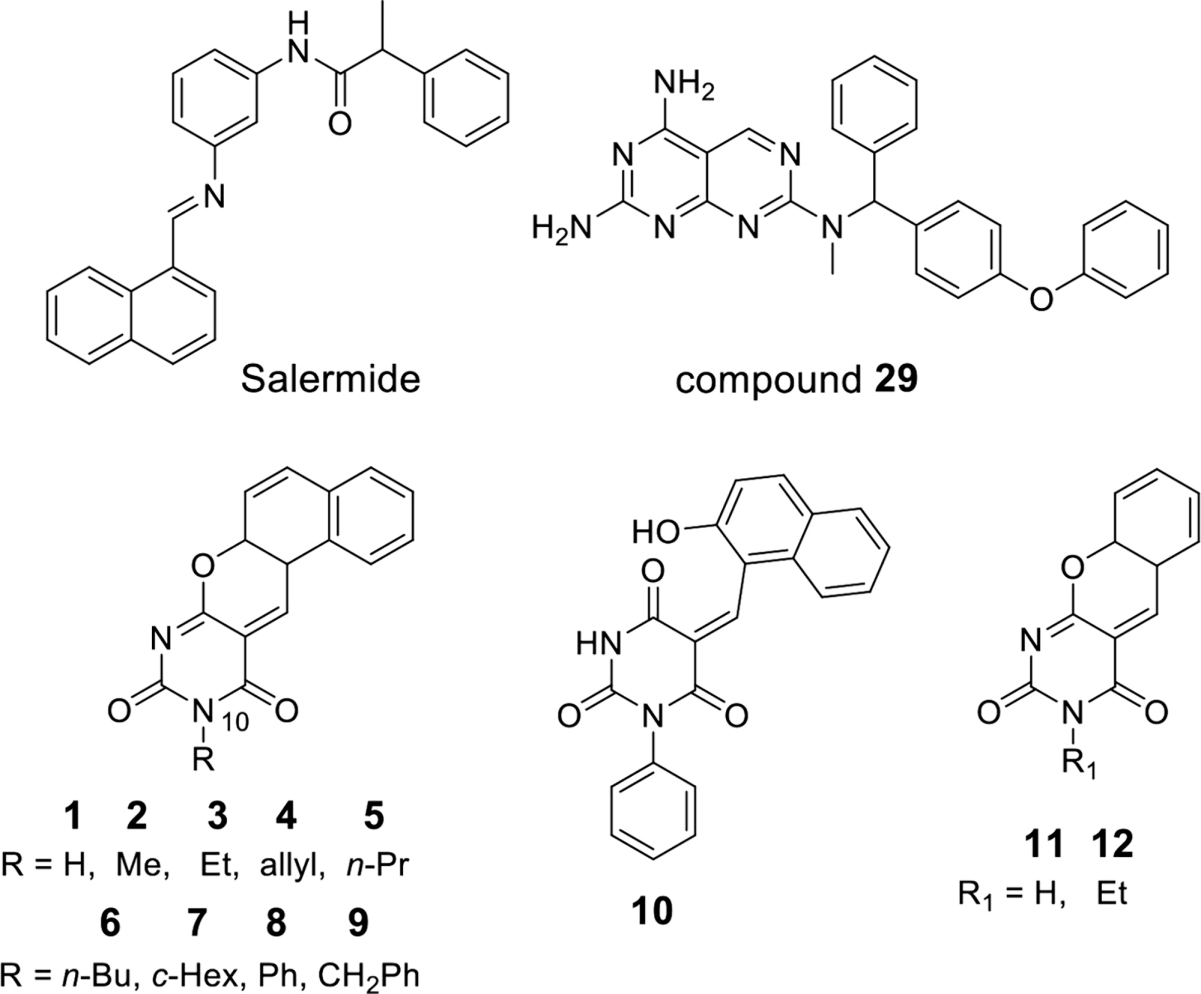
Sirt inhibitors tested as antischistosomal agents.

During our research on small-molecule modulators of epigenetic targets, we identified and described several chemically distinct inhibitors and activators of human sirtuins with potential anticancer activity.^35–56^ Among these, benzodeazaoxaflavins (BDFs) emerged as some of the most potent, inhibiting hSirt1/2 at low micromolar concentrations and reducing proliferation in various cancer cell lines, including colon carcinoma and glioblastoma cancer stem cells.^40,42^

We first improved the synthetic method to obtain most of the BDFs analogues **1–12** (Figure 1), and we tested them against recombinant *Sm*Sirt2 using a homogeneous, fluorescent-based *in vitro* assay with (*Z*)-(Ac)­Lys-7-amino-4-methylcoumarin (ZMAL) as the substrate.

Subsequently, we screened the same compounds against Liberian *S. mansoni* newly transformed schistosomula (NTS) and adult worm pairs. Next, the most promising compounds were investigated on adult worm couples of the Puerto Rican *S. mansoni* strain to assess their effects on viability, worm pairing, egg production, maturation, and morphology of worm organs. Finally, we established a functional link between *Sm*Sirt2 deacetylation inhibition and the observed phenotypes through Western blot analysis of worm extracts, detecting changes in histone acetylation levels in treated samples.

## Results

### Effects of Compounds **1–12** against *Sm*Sirt2

#### Chemistry

Compounds **8**,^40^ **10**,^40^ **11**,^57^ and **12** ^42^ were synthesized according to previously reported procedures. Compounds **1–7** and **9** were prepared by reacting the proper barbituric acid derivatives (prepared as described in literature^42^) with the commercially available 2-hydroxy-1-naphthaldehyde in isopropanol using a microwave-assisted method that reduced the reaction time and afforded higher yields compared to previously reported procedures (Scheme 1).^40,42^

**Scheme 1:**
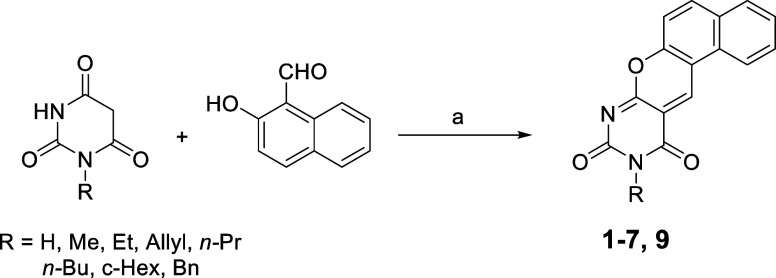
Microwave-Assisted Synthesis of **1–7**, **9** ^a^

a Conditions: (a) 2-propanol, microwave heating at 130 °C, 30–60 min.

#### Biochemistry

The BDF4 compounds **1–12** were tested for their inhibitory activity against *Sm*Sirt2 using a fluorescence-based assay^58^ with ZMAL as the substrate. Their activity against hSirt2 has been previously reported.^40,42^

As shown in Table 1, BDF4 derivatives bearing a hydrogen (1), methyl (2), or phenyl (**8**) group at the N10 position exhibited the highest potency against *Sm*Sirt2. These were followed by compounds **5,** **6,** and **9**, which carry *n*-propyl, *n*-butyl, and benzyl substituents at N10, respectively, and showed inhibitory activity in the low micromolar range. The N10 allyl- (**4**) and cyclohexyl- (**7**) substituted compounds demonstrated lower potency.

**Table 1:** Effect of Compounds **1–12** on *Sm*Sirt2 Activity

		IC_50_ (μM) or % inhibition at 50 μM	
lab code	compd	*Sm*Sirt2	hSirt2
MC2183	**1**	3.8 ± 0.3	>50^a^
MC2358	**2**	2.3 ± 0.1	11.2 ± 0.3^b^
MC2319	**3**	12.6 ± 2.2	30.0 ± 1.2^b^
MC2344	**4**	*53.9%*	10.8 ± 0.3^b^
MC2336	**5**	8.0 ± 0.7	12.5 ± 0.5^b^
MC2333	**6**	7.8 ± 0.8	16.9 ± 0.8^b^
MC2346	**7**	*24.6%*	58.5 ± 2.9^b^
MC2141	**8**	5.3 ± 0.4	12.3 ± 0.5^b^
MC2345	**9**	7.3 ± 0.5	15.9 ± 0.6^b^
MC2139	**10**	*3.5%*	*0.7%* ^a^
MC2184	**11**	*31.0%*	*16.3%* ^a^
MC2852	**12**	*44.6%*	22.5 ± 0.9^b^

a Ref 40.

b Ref 42.

Most potent compounds showed limited selectivity (2- to 5-fold) toward *Sm*SIRT2 over the human isoform, except for compound **1**, which was selectively active against the parasite enzyme. The *N*-phenylbarbiturate derivative **10** failed to inhibit either *Sm*Sirt2 or hSirt2, while the simplified tricyclic analogs **11** and **12** exhibited low to moderate potency against both enzymes.

### Screening of Sirt2 Inhibitors **1**–**9**, **11**, **12** against NTS and Adult Worms *S. mansoni* of Liberian Strain

All compounds active against the recombinant *Sm*Sirt2 protein (the tetracyclic derivatives **1–9** and the tricyclic analogues **11** and **12**) were tested at the Swiss Tropical and Public Health (Swiss TPH) Institute against NTS at concentrations of 20, 10, 1, and 0.1 μM for 72 h, and their corresponding IC_50_ values were determined as previously described.^26,27^ As shown in Table 2, compounds with allyl, *n*-propyl, phenyl, and benzyl substituents at N10 of the tetracyclic scaffold (compounds **4**, **5**, **8**, and **9**, respectively) exhibited the highest potency, with IC_50_ values around 0.1 μM. These were followed by the N10–H and N10-cyclohexyl derivatives **1** and **7**, which showed IC_50_ values of approximately 0.2 μM. The remaining analogues were less effective, with the N10-*n*-butyl-containing compound **6** being the least potent.

**Table 2:** Reduction of NTS (Liberian Strain) Viability by Sirt2 Inhibitors **1–9**, **11**, **12**

		percentage of reduction of NTS viability, 72 h^a^				
lab code	compd	20 μM	10 μM	1 μM	0.1 μM	**IC_50_ (μM)^b^**
MC2183	**1**	100 (0)	100 (0)	90 (1.7)	16.7 (0)	0.25
MC2358	**2**	85.7 (3.6)	61.5 (3.8)	43.8 (2.1)	20.8 (0)	1.50
MC2319	**3**	100 (0)	100 (0)	40 (0)	ND^c^	1.03
MC2344	**4**	100 (0)	100 (0)	100 (0)	20 (0)	0.12
MC2336	**5**	100 (0)	100 (0)	100 (0)	24 (0)	0.12
MC2333	**6**	34 (1)	ND	ND	ND	ND
MC2346	**7**	100 (0)	100 (0)	67.7 (0)	43.8 (2.1)	0.21
MC2141	**8**	100 (0)	100 (0)	100 (0)	22 (1)	0.12
MC2345	**9**	100 (0)	100 (0)	100 (0)	36 (2)	0.11
MC2184	**11**	96 (2)	32.7 (1)	29.2 (0)	25 (0)	1.72
MC2852	**12**	100 (0)	100 (0)	40 (0)	ND	1.03

a The number of replicates is at least *n* = 2; SD is reported in brackets.

b IC_50_, compound concentration that inhibits 50% of the viability of parasites.

c ND, not determined.

Following the NTS screening, the Sirt2 inhibitors **1–9**, **11**, and **12** (excluding compound **6**) were tested against adult *S. mansoni* (Liberian strain) worm pairs. Parasites were treated with compounds at a concentration of 20 μM, and viability was assessed at 72 h as previously described.^26,27^ Those that exhibited a ≥50% reduction in worm viability at 20 μM were further tested at 10 μM, and, if applicable, at 1 μM to determine their IC_50_ values (Table 3).

**Table 3:** Effects of Sirt2 Inhibitors **1–5**, **7–9**, **11**, and **12** on *S. mansoni* (Liberian Strain) Adult Worm Viability Reduction

		**adult worms’ percentage of viability reduction,**72 h^a^			
lab code	compd	20 μM	10 μM	1 μM	**IC_50_ (μM)^b^**
MC2183	**1**	85.7 (7.2)	27.5 (2)	ND^c^	>10
MC2358	**2**	82.1 (3.6)	37.3 (3.9)	ND	>10
MC2319	**3**	80.3 (5.4)	49 (0)	ND	>10
MC2344	**4**	94.4 (0)	58.6 (0)	40.7 (3.7)	2.03
MC2336	**5**	91.7 (2.8)	58.6 (0)	25.9 (3.7)	3.35
MC2346	**7**	100 (0)	100 (0)	23.9 (0)	1.24
MC2141	**8**	97.2 (2.8)	37.9 (0)	ND	>10
MC2345	**9**	61.2 (5.6)	58.6 (6.9)	55.6 (0)	<1
MC2184	**11**	49.9 (0)	ND	ND	>20
MC2852	**12**	75.0 (3.6)	41.2 (0)	ND	>10

a The number of replicates is at least *n* = 2; SD is reported in brackets.

b IC_50_, compound concentration that inhibits 50% of the viability of parasites.

c ND, not determined.

Among the tested compounds, **4, 5, 7,** and **9** emerged as the most potent. Specifically, compounds **4, 5, 7,** and **9** reduced worm viability by more than 50% at 10 μM, and 9 achieved the same effect at just 1 μM. Notably, the N10-benzyl-substituted compound **9** demonstrated the highest efficacy with an IC_50_ <1 μM.

### Effects of Selected Sirt2 Inhibitors on the Viability of Adult *S. mansoni* (Puerto Rican Strain) Worm Pairs, Egg Production, and Organ Maturation

To investigate the phenotypic effects of BDF4-derived Sirt2 inhibitors on adult worm pairs, parasites were treated with compounds **1, 4, 5**, and **7–9** at concentrations of 20, 10, and 1 μM at the Schistodiscovery unit of the CNR-IBBC in Monterotondo (Rome), Italy. Worm viability was assessed daily by scoring each pair on a scale from 3 (no effect) to 0 (severe effects) for 72 h, based on multiple phenotypic features including plate attachment, movement, color, gut peristalsis, and tegument integrity, as previously described (Figure 2).^59,60^

**Figure 2:**
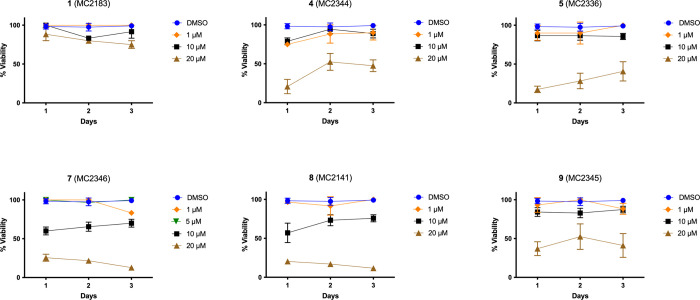
Adult schistosome pairs (Puerto Rican strain) viability. Dose–response curve of the inhibitors on adult schistosome worm pairs. DMSO (vehicle) was used as negative control (100% viability); the indicated compounds were assayed at 1 μM (diamond ), 10 μM (square), and 20 μM (triangle). Data are expressed as a % severity score (viability) relative to DMSO. Each point represents the average ± standard deviation of three-four independent experiments.

Among the tested compounds, **7,** **8**, and **9** at 20 μM were the most potent, inducing 70–80% mortality in adult *S. mansoni*worm pairs within 24 h; with complete lethality by 72 h. Compounds **7** and **8** also exhibited partial efficacy at 10 μM, with a reduction of 40–30 and 40% viability, respectively (Figure 2). Compound **1** showed a reduction of only 20% at 72 h, compounds **4** and **5** of approximately 80% a 24 h; however, the parasites gradually recovered with a viability at 72 h of 50 and 40%, respectively. Under the same conditions, salermide, an inhibitor of hSirt1/2 previously shown to induce apoptosis in various cancer cell lines^34^ and schistosomula,^33^ did not affect adult worm viability after 72 h in our study.

Previous studies have reported that Sirt1 and Sirt2 inhibitors disrupt worm pairing and egg laying.^33^ Therefore, we monitored these parameters. All compounds, except **1**, disrupted worm pairing (Table 4). Regarding the impacts on egg laying, the effect was minimal at sublethal concentrations (10 μM) and more pronounced at lethal doses (Table 4, Figure S2).

**Table 4:** Impact of Sirt2 Inhibitors on *S. mansoni* (Puerto Rican) Worm Pairing and Egg Laying^a^

			% worm pairing			% egg laying		
lab code	compd	[μM]	24 h	48 h	72 h	24 h	48 h	72 h
MC2183	**1**	1	100	100	100	79	90	116
		10	100 (0)	100 (0)	100 (0)	93 (27)	97 (14)	122 (14)
		20	100 (0)	100 (0)	100 (0)	56 (22)	75 (19)	109 (47)
MC2344	**4**	1	100	100	100	72	68	95
		10	90 (14)	90 (14)	90 (14)	61(6)	82 (12)	79 (19)
		20	0 (0)	20 (0)	60 (0)	11 (6)	7 (5)	9 (39)
MC2336	**5**	1	100	100	100	104	94	144
		10	90 (14)	100 (14)	100 (14)	55 (15)	73 (33)	75 (7)
		20	0 (0)	10 (14)	0 (0)	12 (16)	11 (14)	13 (10)
MC2346	**7**	1	100	100	100	75	66	75
		10	100 (0)	100 (0)	100 (0)	32 (4)	46 (6)	44 (10)
		20	0 (0)	0 (0)	6 (11)	14 (12)	9 (8)	6 (59
MC2141	**8**	1	100	100	100	106	91	113
		10	33 (23)	66 (23)	80 (20)	25 (16)	43 (17)	51 (19)
		20	0 (0)	0 (0)	20 (36)	9 (8)	4 (4)	3 (3)
MC2345	**9**	1	100	100	100	98	98	126
		10	80 (20)	93 (11)	100 (0)	72 (74)	71 (36)	67 (28)
		20	6 (11)	20 (30)	20 (30)	11 (7)	8 (5)	7 (6)

a The data of at least three independent experiments (except for the 1 μM treatments that represent a single experiment) are presented as percentage of control values (DMSO). The samples treated with DMSO at all time points and with the Sirt2 inhibitors at time 0 showed 100% of worm pairing. Average values ± SD (in brackets) are shown.

To exclude a direct effect of the inhibitors on the viability or development of *in vitro* laid eggs (IVLEs), eggs produced in the first 48 h by worm couples were collected and treated for 3 days with either the vehicle (DMSO) or sirtuin inhibitors. The eggs were then counted and classified by viability and maturation stage through microscopic observation following the Vogel and Prata system.^61,62^ No differences were observed between the treated and control groups. Representative images are shown in Figure S1.

To further characterize the phenotypic alterations, carmine-red staining followed by confocal laser scanning microscopy (CLSM) was performed as previously described (Figure 3).^63^ For each compound, three to five worm pairs were analyzed, with imaging of ovaries, ootype, vitellarium, testes, and gut. Treated worms exhibited less organized ovaries, with a reduction in size and number of both immature (anterior) and mature (posterior) oocytes. Degeneration of mature oocytes (mo) and increased black inclusions were observed. The ootype appeared empty or contained malformed eggs or fragments. The vitellarium retained its morphology in all samples, but reduced cellularity and signs of cell degeneration were noted in worms treated with the compounds at 10 μM concentration. Moreover, the Sirt2 inhibitors caused gut dilation, with a decrease in the number and/or alterations of surface amplification and lumen epithelial thinning with degradation of the gastrodermis. Carmine-red aggregates were also detected in the gut lumen. In testis, cellularity and cell heterogeneity decreased following treatment. These analyses were performed at a sublethal concentration (10 μM) to avoid morphological changes due to worm separation. All effects were more pronounced on day 6 (10 μM) (Figure S2). As expected, ovary and vitellarium structures were completely disrupted in unpaired females at day 6 (Figure S3), in agreement with earlier observations for hSirt1/2 inhibitors.^33^

**Figure 3:**
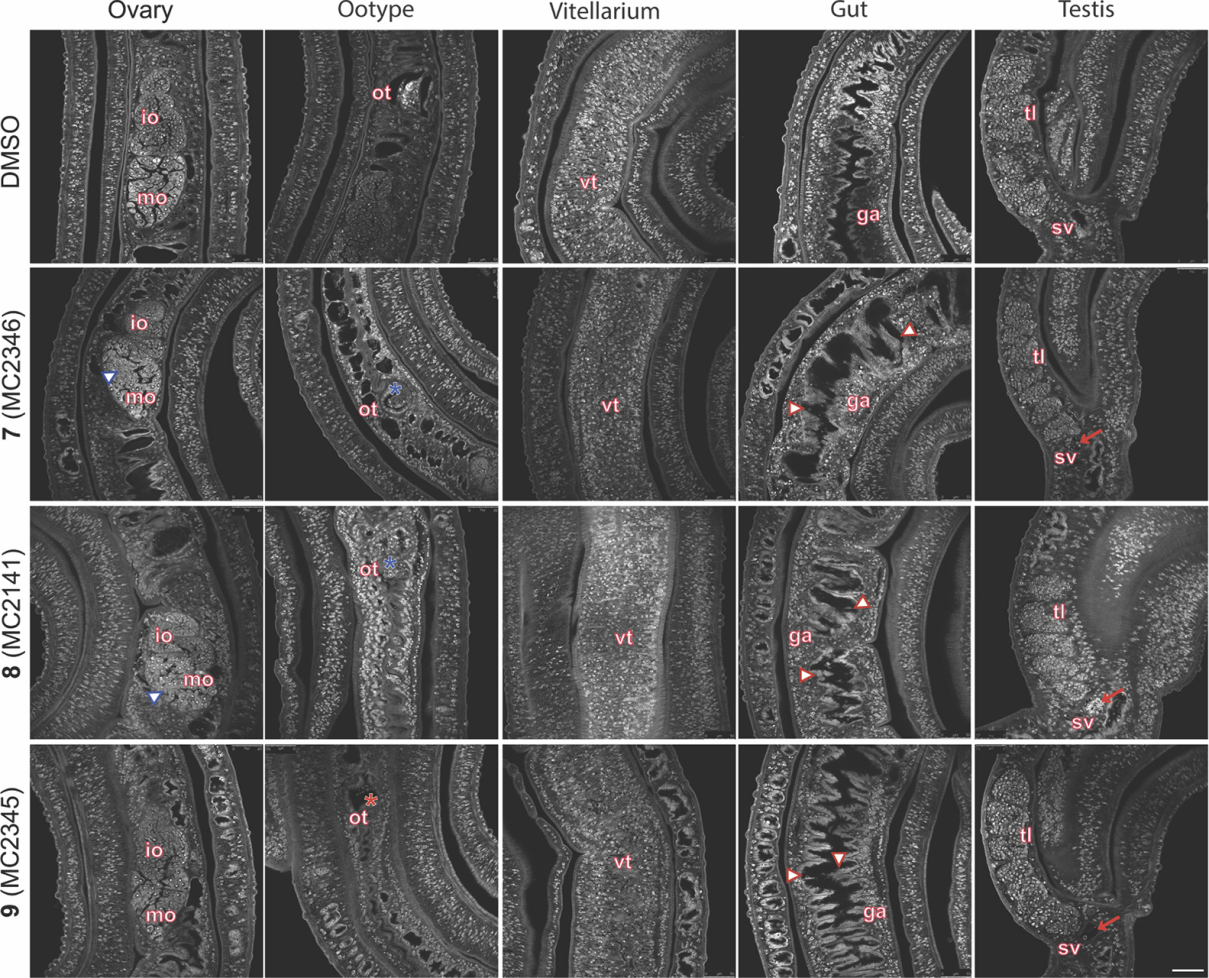
Confocal microscopy of carmine-red stained *S. mansoni* (Puerto Rican strain) adult worm pairs treated with Sirt2 inhibitors. The images are representative of the observation of three to four worm pairs treated with either vehicle (DMSO) or the specified inhibitors at a concentration of 10 μM for 72 h. The ovary, ootype, vitellarium, gut, and testis are all visible in the images. Immature oocytes (io), mature oocytes (mo), ootype (ot), gastrodermis (ga), testicular lobes (tl), and seminal vesicles (sv) are labeled. In the images degenerated, mo are marked with blues contoured triangles, deformed eggs or egg fragments in the ootype are denoted by blue asterisks, and an empty ootype is indicated by a red asterisk. Ga alterations in the gut are highlighted with red countered triangles, while spermatozoa accumulation in the sv is shown with red arrows. The scale bar measures 50 μm.

### Effects of Selected Sirt2 Inhibitors on Histone Acetylation

To evaluate the impact of Sirt2 inhibitors on protein acetylation, cytosolic and histone-enriched protein fractions from treated *S. mansoni* (Puerto Rican strain) worm pairs (24 and 48 h) were analyzed by Western blotting. An increase in acetylation of histone H3 (pan-acetylation) but not histone H4 (pan-acetylation), was consistently observed in samples treated with compounds **7, 8**, and **9** for 48 h (Figure 4 and Figure S4). Total lysine acetylation was similar in control and Sirt2 inhibitor-treated samples (Figure S4).

**Figure 4:**
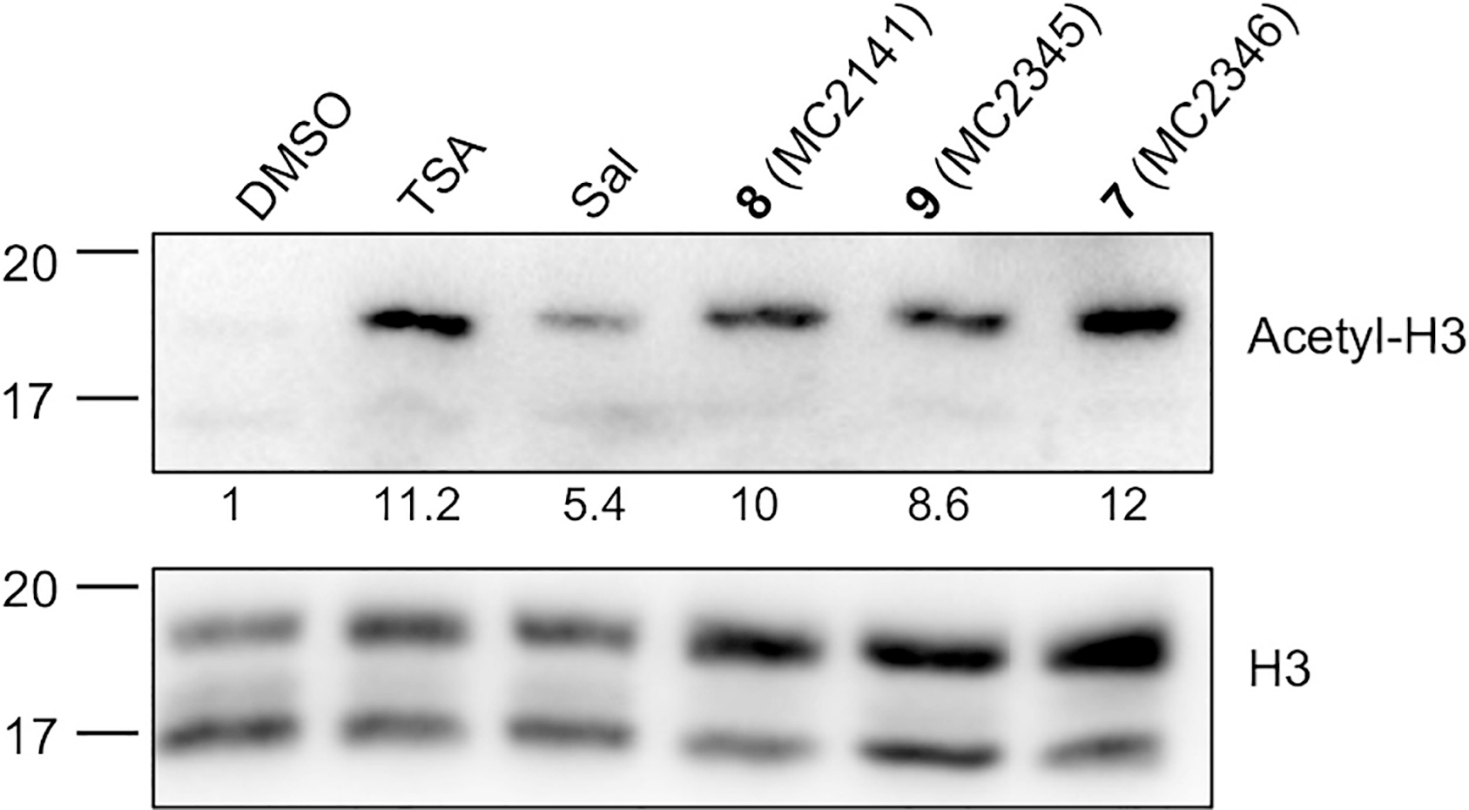
Effects of selected Sirt2 inhibitors on histone H3 acetylation. Representative immunoblots of histone-enriched protein fractions extracted from adult *S. mansoni* worm pairs are shown. The worms were treated with the indicated compounds. DMSO (vehicle), TSA (1 μM, 24 h), and salermide (Sal 25 μM, 48 h) were used as controls. Sirt2 inhibitors **7–9** were used at a concentration of 10 μM for 48 h. The total H3 signal was used for normalization. The quantitation of acetyl-H3 (pan-acetylated) relative to the vehicle-treated sample is displayed.

### Effects of Selected Sirt2 Inhibitors on Cytochrome c Release

To determine if the Sirt2 inhibitors trigger apoptosis through the cytochrome c pathway, we examined cytochrome c release from mitochondria. Immunoblotting of cytosolic fractions revealed increased levels of cytochrome c in *S. mansoni* (Puerto Rican strain) worms treated for with TSA, salermide, and the BDF4-derived inhibitors **7–9** for 48 h (Figure 5). Salermide is known to induce tumor-specific apoptosis in various of human cancer cell lines^34^ and cause DNA fragmentation, as shown by TUNEL assay, in schistosomula after 48 h of treatment (10–20 μM).^33^

**Figure 5:**
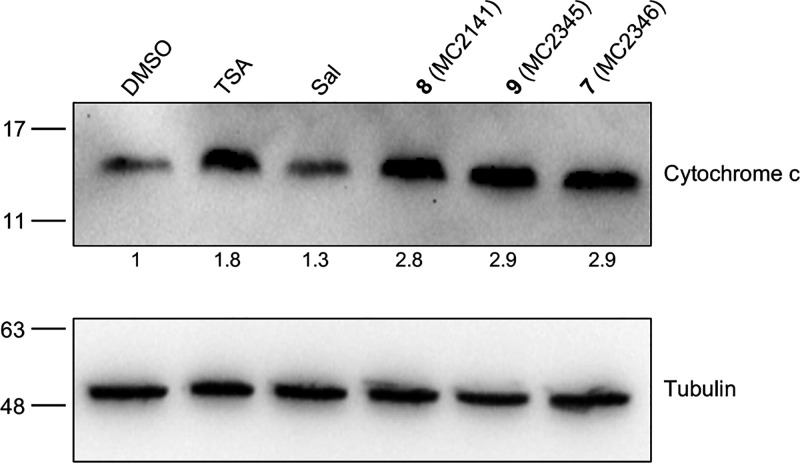
Effects of selected Sirt2 inhibitors on cytosolic cytochrome c expression. Representative immunoblots of cytosolic protein fractions extracted from *S. mansoni* adult worm pairs are shown. The worms were treated with the indicated Sirt2 inhibitors (10 μM) for 48 h. DMSO (vehicle), TSA (1 μM, 24 h), a HDAC pan-inhibitor, and salermide (Sal 25 μM, 48 h), a Sirt1/Sirt2 inhibitor, were used as controls. The α-tubulin signal was used for normalization. Quantitation of cytochrome c relative to the vehicle-treated sample is shown.

### Activity of the Hit Compounds on Mammalian Cell Lines

The cytotoxicity of selected compounds was evaluated on two different cell lines, murine L929 and human BJ fibroblasts, at 72 h using MTT viability assays, as described previously.^64^ All three compounds demonstrated a safe profile on both cell lines with an IC_50_ greater than 50 μM. The negative control used was DMSO (vehicle), while gambogic acid served as the positive control (Figure 6).

**Figure 6:**
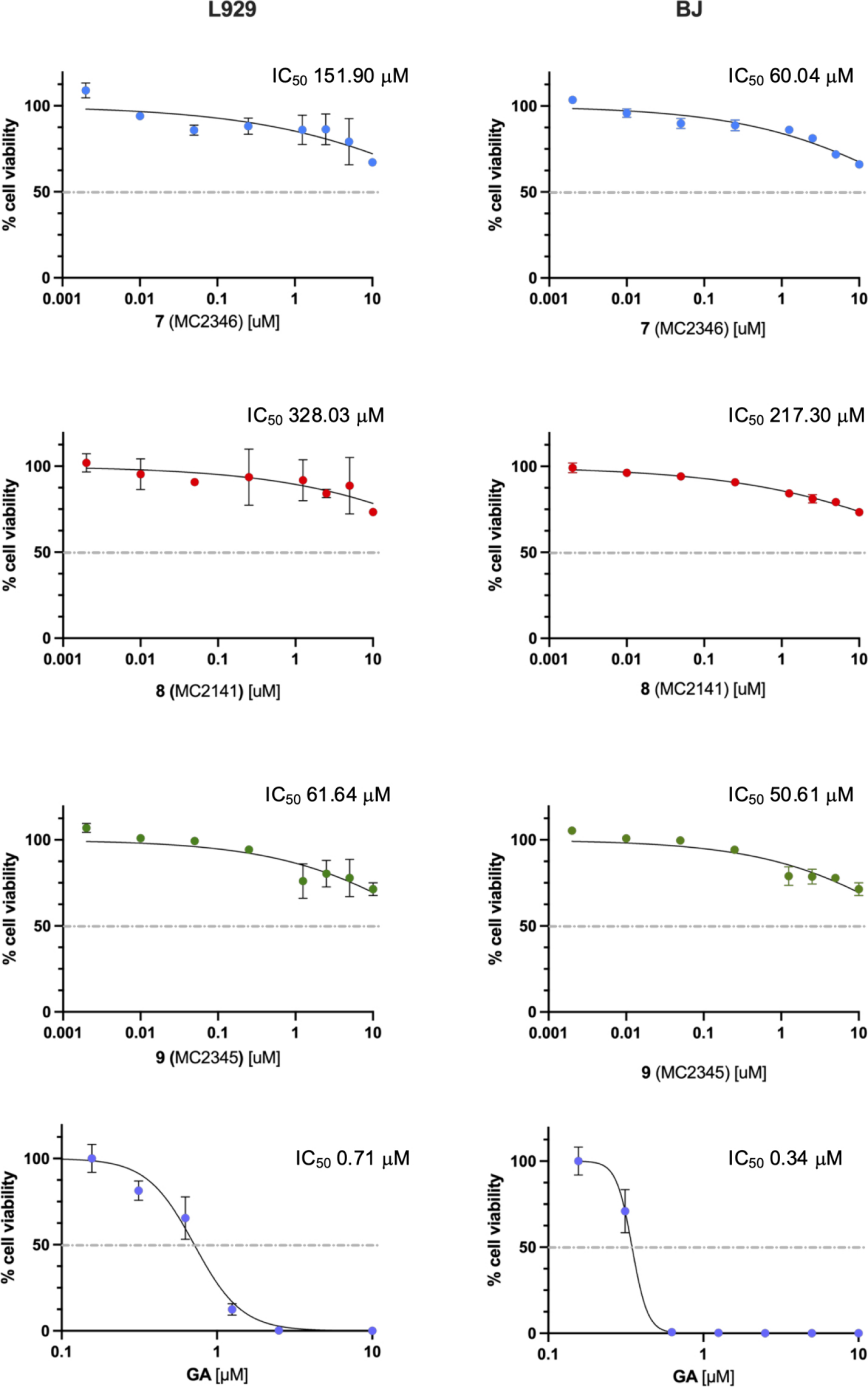
Cell viability MTT assays. The dose–response curves for compounds **7** (MC2346), **8** (MC2341), and **9** (MC2345) on BJ and L929 fibroblast cell lines are presented. DMSO (vehicle) and GA (gambogic acid) were used as negative control (100% viability) and positive controls, respectively. Each point on the graph represents the average ± standard deviation of three (L929) or two (BJ) independent experiments. The *x*-axis (anti-log scale) shows the compound concentrations used in the assays. The IC_50_ calculated using GraphPad Prism is also included.

## Discussion

This study reinforces the potential of targeting parasite-specific epigenetic regulators, particularly sirtuins, as an innovative therapeutic strategy for schistosomiasis. Our results demonstrate that benzodeazaoxaflavin (BDF4)-based compounds inhibit the *Sm*Sirt2 enzyme. Compounds **1**, **2**, and **8** showed inhibitory activity in the low micromolar range followed by compounds **5**, **6**, and **9**. Compounds **4**, **7**, **11**, and **12** demonstrated lower potency *in vitro*. Among the Sirt2 inhibitors exhibiting antischistosomal activity in two different *S. mansoni* strains, compounds **7–9** (MC2346, MC2141, and MC2345) emerged as the most effective ones. Since compound **7** demonstrated low potency *in vitro* in the SmSirt2 inhibitory assay, we cannot exclude the possibility that it targets other *S. mansoni* proteins or Sirt isoenzymes *in vivo.* However, we should consider the complex regulation of Sirt2 enzymatic activity *in vivo*, including the enzyme’s conformational plasticity, interactions with several substrates and other binding partners, and functional consequences of posttranslational modifications such as phosphorylation. A review by Bernhard et al. discusses the complexities of Sirt2 activity modulation.^65^ Therefore, establishing a clear correlation between *in vitro* and *in vivo* studies of Sirt2 inhibitors is challenging. Further investigation into the specificity of compound **7** is required.

BDF4 derivatives showed similar potency against both schistosomula and adult worm stages. Unlike what was previously observed by us with LSD1 inhibitors, where there was an approximately linear correlation between anti-LSD1 potency and schistosome viability reduction,^66^ and similarly to findings reported for some *Sm*HDAC8 inhibitors^27,67^ and a series of pyrimido­[4,5-*d*]­pyrimidines with anti-*Sm*Sirt2 activity,^30^ the BDF4 derivatives described here show only a limited correlation between *Sm*Sirt2 inhibition and *in vitro* antiparasitic activity.

The investigation of BDF4 compounds on adult parasites for selected compounds was conducted in two different laboratories located at the Swiss TPH (Allschwil) and at the CNR-IBBC Monterotondo (Rome). While differences were observed for some compounds and the concentrations required to kill adult parasites *in vitro*, the study allowed the selection of three compounds with a common interesting structure, active on two stages of *S. mansoni* as promising candidates for the treatment of schistosomiasis.

Differences in assay design, worm culture conditions, particularly the use of culturing in the presence of fetal bovine serum, score assignment, and the parasite strain used, have previously been considered to explain interassay variability in compounds screening on *S. mansoni* in different laboratories.^68,69^ Interestingly, literature data suggest that the laboratory Puerto Rican strain, like the Belo Horizonte strain, has higher virulence than the Liberian strain. Moreover, differences in host–parasite interactions, immune responses, and organ pathology in mice have also been reported with laboratory Puerto Rican and Liberian strains. The Puerto Rican strain induced stronger liver fibrosis, higher egg loads, and more pronounced tissue damage compared to the Liberian strain.^70^ For these reasons, studying compounds in different *S. mansoni* strains could help to identify the most promising hits.

Importantly, all Sirt2 inhibitors characterized, except **1**, disrupted worm pairing. *Schistosomes* are the only platyhelminths with separate sexes, and female sexual maturation requires continuous pairing with a male. It is known that adult worm pairs in culture cease viable egg production after a few days,^71,72^ with a faster decline in unpaired females.^73^ Separated females surgically implanted into a host stop laying eggs and regress to an immature state, although this regression is reversible upon repairing.^4,6,74^ This regression is largely due to involution of the vitellarium, which produces eggshell components and nutrients for the embryo.^73^ Therefore, a reduction in egg-laying in treated worms was expected for compounds affecting pairing.

The observed phenotypic effects—reduced worm viability, disrupted pairing, and decreased egg production, associated also to damage of reproductive organs—highlight the central role of *Sm*Sirt2 in parasite survival and reproduction.

Furthermore, the correlation between increased histone H3 acetylation and cytochrome c release in treated worms confirms that *Sm*Sirt2 inhibition disrupts essential cellular processes and activates apoptotic pathways.

## Conclusions

In conclusion, we explored a new class of compounds—benzodeazaoxaflavins (BDF4)—about their ability to inhibit *Sm*Sirt2, a parasite enzyme involved in epigenetic regulation. We identified three effective compounds, **7**–**9** (MC2346, MC2141 and MC2345), which showed activity against both schistosomula and adult stages of Liberian and Puerto Rican strains of *Schistosoma mansoni*. These compounds impaired the viability of the worms and egg production and caused morphological damage in reproductive and gut tissues. Importantly, their effects were linked to increased histone acetylation and the activation of apoptosis pathways by cytochrome c release. These findings support further preclinical development of *Sm*Sirt2 inhibitors as next-generation treatments for schistosomiasis, particularly considering the threat for PZQ resistance occurrence.

Future studies should focus on the efficacy of the compounds on other *Schistosoma* species, *in vivo* efficacy, pharmacokinetics, and safety profiling to validate these inhibitors for potential clinical application.

## Experimental Section

### Chemistry

Melting points were determined on a Buchi 530 melting point apparatus and are uncorrected. ^1^H NMR and ^13^C NMR spectra were recorded at 400 MHz on a Bruker AC 400 spectrometer. Chemical shifts are reported in δ (ppm) units relative to the internal reference tetramethylsilane (Me_4_Si). Microwave-assisted reactions were performed with a Biotage Initiator (Uppsala, Sweden) high-frequency microwave synthesizer working at 2.45 GHz, fitted with a magnetic stirrer and sample processor; reaction vessels were Biotage microwave glass vials sealed with an applicable cap; and temperature was controlled through the internal IR sensor of the microwave apparatus. Low-resolution mass spectra of final compounds were recorded on an API-TOF Mariner by Perspective Biosystem (Stratford, Texas, USA); samples were injected by a Harvard pump using a flow rate of 5–10 μL/min, infused in the electrospray system. All compounds were routinely checked by TLC and ^1^H NMR; all final compounds were also checked by ^13^C NMR. TLC was performed on aluminum-backed silica gel plates (Merck DC, Alufolien Kieselgel 60 F_254_) with spots visualized by UV light. All solvents were of reagent grade and, when necessary, were purified and dried by standard methods. Concentration of solutions after reactions and extractions involved the use of a rotary evaporator operating at a reduced pressure of ca. 20 Torr. The purity of compounds **1**–**7** and **9** was analyzed by elemental analysis. Analytical results are within ±0.40% of the theoretical values (Table S1). Organic solutions were dried over anhydrous sodium sulfate. All chemicals were purchased from Sigma-Aldrich s.r.l, Milan (Italy), or from TCI Europe N.V., Zwijndrecht (Belgium), and were of the highest purity.

#### General Procedure for the Preparation of Final Compounds **1–7** and 9

A mixture of the appropriately substituted barbituric acid (1.0 mmol, 1.0 equiv) and commercially available 2-hydroxy-1-naphthaldehyde (1.1 mmol, 1.1 equiv) was placed in a microwave glass vial and dissolved in 2-propanol (1 mL). The reaction mixture was heated under microwave irradiation at 130 °C for 30 min to 1 h, until complete consumption of the starting material (monitored by TLC). The resulting hot suspension was filtered off, and the crude yellow solid was purified by recrystallization from a 9:1 (v/v) mixture of acetic acid and acetic anhydride, affording compounds **1**–**7** and **9** in very high yields.

#### 7-Oxa-8,10-Diazabenzo­[*a*]­anthracene-9,11-dione (**1**, MC2183)

Yellow solid; mp >260 °C; yield 91%. ^1^H NMR (DMSO) δ 7.70–8.83 (m, 6H, C_1–6_-*H* polycyclic system), 9.52 (s, 1H, C_12_-*H* polycyclic system), 11.44 (s, 1H, N*H*). ^13^C NMR (DMSO) δ 117.7, 117.9, 120.2, 122.5, 123.7, 127.1, 128.2, 128.7, 128.8, 130.1, 131.9, 150.5, 159.6, 164.2, 164.9 ppm. Anal. (C_15_H_8_N_2_O_3_) C, H, N. MS (ESI), *m*/*z*: 264 [M + H]^+^.

#### 10-Methyl-9*H*-benzo­[5,6]­chromeno­[2,3-*d*]­pyrimidine-9,11­(10H)-dione (**2**, MC2358)

Yellow solid; mp >260 °C; yield 93%. ^1^H NMR (DMSO) δ 3.47 (s, 3H, NC*H*_3_), 7.73–7.84 (m, 3H, C_1–6_-*H* polycyclic system), 8.15 (m, 1H, C_1–6_-*H* polycyclic system), 8.50 (m, 1H, C_1–6_-*H* polycyclic system), 8.85 (m, 1H, C_1–6_-*H* polycyclic system), 9.59 (s, 1H, C_12_-*H* polycyclic system). ^13^C NMR (DMSO) δ 26.3, 115.5, 116.3, 117.7, 122.3, 123.6, 126.8, 128.7, 130.1, 130.3, 150.5, 158.8, 160.3 ppm. Anal. (C_16_H_10_N_2_O_3_) C, H, N. MS (ESI), *m*/*z*: 279 [M + H]^+^.

#### 10-Ethyl-9*H*-benzo­[5,6]­chromeno­[2,3-*d*]­pyrimidine-9,11­(10H)-dione (**3**, MC2319)

Yellow solid; mp >260 °C; yield 90%. ^1^H NMR (DMSO) δ 1.15 (t, 3H, NCH_2_C*H*_3_), 3.89 (q, 2H, NC*H*_2_CH_3_), 7.70–7.81 (m, 3H, C_1–6_-*H* polycyclic system), 8.14 (m, 1H, C_1–6_-*H* polycyclic system), 8.49 (m, 1H, C_1–6_-*H* polycyclic system), 8.83 (m, 1H, C_1–6_-*H* polycyclic system), 9.58 (s, 1H, C_12_-*H* polycyclic system). ^13^C NMR (DMSO) δ 11.8, 38.5, 115.5, 116.3, 117.7, 122.3, 123.6, 126.8, 128.7, 130.1, 130.3, 150.5, 158.8, 160.0, 160.4 ppm. Anal. (C_17_H_12_N_2_O_3_) C, H, N. MS (ESI), *m*/*z*: 293 [M + H]^+^.

#### 10-Allyl-9*H*-benzo­[5,6]­chromeno­[2,3-*d*]­pyrimidine-9,11­(10H)-dione (**4**, MC2344)

Yellow solid; mp >260 °C; yield 96%. ^1^H NMR (DMSO) δ 4.48 (s, 2H, NC*H*_2_), 5.08–5.15 (m, 2H, C-*H* vinyl sytem), 5.85 (m, 1H, C-*H* vinyl sytem), 7.72–7.85 (m, 3H, C_1–6_-*H* polycyclic system), 8.14 (m, 1H, C_1–6_-*H* polycyclic system), 8.50 (m, 1H, C_1–6_-*H* polycyclic system), 8.83 (m, 1H, C_1–6_-*H* polycyclic system), 9.59 (s, 1H, C_12_-*H* polycyclic system). ^13^C NMR (DMSO) δ 45.5, 115.5, 116.3, 117.4, 117.7,122.3, 123.6, 126.8,128.7, 130.1, 130.3, 132.2, 150.5, 158.8, 160.4 ppm. Anal. (C_18_H_12_N_2_O_3_) C, H, N. MS (ESI), *m*/*z*: 305 [M + H]^+^.

#### 10-Propyl-9*H*-benzo­[5,6]­chromeno­[2,3-*d*]­pyrimidine-9,11­(10H)-dione (**5**, MC2336)

Yellow solid; mp >260 °C; yield 92%. ^1^H NMR (DMSO) δ 0.87 (t, 3H, C*H*_3_), 1.58 (m, 2H, C*H*_2_CH_3_), 3.83 (t, 2H, NC*H*_2_CH_2_CH_3_), 7.72–7.83 (m, 3H, C_1–6_-*H* polycyclic system), 8.14 (m, 1H, C_1–6_-*H* polycyclic system), 8.49 (m, 1H, C_1–6_-*H* polycyclic system), 8.83 (m, 1H, C_1–6_-*H* polycyclic system), 9.57 (s, 1H, C_12_-*H* polycyclic system). ^13^C NMR (DMSO) δ 10.8, 19.9, 46.16, 115.5, 116.3, 117.7, 122.3, 123.6, 126.8, 128.5, 128.7, 128.8, 130.1, 130.3, 150.5, 158.5, 160.0, 160.4 ppm. Anal. (C_18_H_14_N_2_O_3_) C, H, N. MS (ESI), *m*/*z*: 307 [M + H]^+^.

#### 10-Butyl-9*H*-benzo­[5,6]­chromeno­[2,3-*d*]­pyrimidine-9,11­(10H)-dione (**6**, MC2333)

Yellow solid; mp 233–235 °C; yield 91%. ^1^H NMR (DMSO) δ 0.88 (t, 3H, NCH_2_CH_2_CH_2_C*H*_3_), 1.30 (m, 2H, NCH_2_CH_2_C*H*_2_CH_3_), 1.54 (m, 2H, NCH_2_C*H*_2_CH_2_CH_3_), 3.87 (t, 2H, NC*H*_2_CH_2_CH_2_CH_3_), 7.72–7.86 (m, 3H, C_1–6_-*H* polycyclic system), 8.14 (m, 1H, C_1–6_-*H* polycyclic system), 8.49 (m, 1H, C_1–6_-*H* polycyclic system), 8.83 (m, 1H, C_1–6_-*H* polycyclic system), 9.57 (s, 1H, C_12_-*H* polycyclic system). ^13^C NMR (DMSO) δ 13.8, 19.4, 29.0, 44.1, 115.5, 116.3, 117.7, 122.3, 123.6, 126.8, 128.7, 130.1, 130.3, 150.5, 158.8, 160.0, 160.4 ppm. Anal. (C_19_H_16_N_2_O_3_) C, H, N. MS (ESI), *m*/*z*: 321 [M + H]^+^.

#### 10-Cyclohexyl-9*H*-benzo­[5,6]­chromeno­[2,3-*d*]­pyrimidine-9,11­(10H)-dione (**7**, MC2346)

Yellow solid; mp >260 °C; yield 98%. ^1^H NMR (DMSO) δ 1.15–1.31 (m, 4H, C-*H* cyclohexyl system), 1.57–1.80 (m, 4H, C-*H* cyclohexyl system), 2.33 (m, 2H, C-*H* cyclohexyl system), 4.65 (m, 1H, NC*H*), 7.71–7.84 (m, 3H, C_1–6_-*H* polycyclic system), 8.13 (m, 1H, C_1–6_-*H* polycyclic system), 8.47 (m, 1H, C_1–6_-*H* polycyclic system), 8.80 (m, 1H, C_1–6_-*H* polycyclic system), 9.52 (s, 1H, C_12_-*H* polycyclic system). ^13^C NMR (DMSO) δ 24.4, 25.7, 30.6, 63.6, 115.5, 116.3, 117.7, 122.3, 123.6, 126.8, 128.8, 130.1, 130.3, 150.5, 158.2, 159.7, 160.4 ppm. Anal. (C_21_H_18_N_2_O_3_) C, H, N. MS (ESI), *m*/*z*: 347 [M + H]^+^.

#### 10-Benzyl-9*H*-benzo­[5,6]­chromeno­[2,3-*d*]­pyrimidine-9,11­(10H)-dione (**9**, MC2345)

Yellow solid; mp >260 °C; yield 95%. ^1^H NMR (DMSO) δ 5.07 (s, 2H, NC*H*_2_), 7.21–7.33 (m, 5H, C-*H* benzyl system), 7.70–7.85 (m, 3H, C_1–6_-*H* polycyclic system), 8.13 (m, 1H, C_1–6_-*H* polycyclic system), 8.48 (m, 1H, C_1–6_-*H* polycyclic system), 8.81 (m, 1H, C_1–6_-*H* polycyclic system), 9.60 (s, 1H, C_12_-*H* polycyclic system). ^13^C NMR (DMSO) δ 46.9, 115.5, 116.3, 117.7, 122.3, 123.6, 126.7, 126.8, 126.9, 128.5, 128.7, 130.1, 130.3, 136.5, 150.5, 158.8, 160.3, 160.4 ppm. Anal. (C_22_H_14_N_2_O_3_) C, H, N. MS (ESI), *m*/*z*: 355 [M + H]^+^.

### Reagents

Chemicals reagents if not otherwise stated were purchased from Merck Life Science Srl (Milan, Italy) or Thermo Fisher Scientific, Italy; tissue culture media reagents, Dulbecco’s modified Eagle’s medium (DMEM), Hepes, l-glutamine, penicillin/streptomycin, and fetal bovine serum (FBS) from Euroclone SpA (Milan, Italy); and Modified Eagle’s medium from Thermo Fisher. Antibiotic–antimicotic (cat. 15240062, Thermo Fisher) was used for the *Schistosome* cultures. The primary monoclonal or polyclonal antibodies used were as follows: histone H3 antibody ab1791 (1.2000) from Abcam; histone H3ac (pan-acetyl) (RRID: AB_2687871) (1:2000) and histone H4ac (pan-acetyl) (RRID: AB_2793201) from Active Motif; cytochrome C (clone 7H8.2C129) (1:2000) from BD Pharmingen; acetylated-lysine (Ac-K2-100) (1:4000) from Cell Signaling Technologies; tubulin (DM1A) (1:5000) from Sigma-Aldrich; goat antimouse and antirabbit IgG (H+L) horseradish peroxidase secondary antibodies from Bio-Rad Laboratories, Italy.

### *In Vitro Sm*Sirt2 Inhibition Assay

For the screening of compounds **1**–**12**, a homogeneous fluorescence-based assay previously described by some of us was used to determine *Sm*Sirt2 activity.^58^ All compounds were initially tested at 50 μM and, for candidates that showed a *Sm*Sirt2 inhibition higher than 50% at this concentration, IC_50_ values have been measured and determined using OriginPro 9.0 G. The absence of eventual assay interference due to trypsin inhibition was confirmed according to the published procedures,^58^ while, to exclude any quenching of the 7-amino-4-methylcoumarin (AMC) signal, 2.5 μL of an AMC solution (prepared from 12.6 mM stock solution in DMSO and diluted with assay buffer; final assay concentration, 10.5 μM) was used instead of ZMAL in the homogeneous assay.

### Ethics Statement

Animal work was approved by the CNR-IBBC Animal Welfare Committee (OPBA) and by the competent authorities of the Italian Ministry of Health, DGSAF, Rome (authorization nos. 336/2018-PR and 667/2023-PR). All experiments were conducted according to the ethical and safety rules and guidelines for the use of animals in biomedical research provided by the relevant Italian laws and European Union’s directives (No. 86/609/EEC and subsequent). For experiments performed at Swiss TPH in Allschwil, approval was given by the veterinary authorities of the Canton Basel-Landschaft (permission no. 545) based on Swiss cantonal and national regulations.

### Newly Transformed *S. mansoni* Schistosomula (NTS) and Adult *S. mansoni* Worm (Liberian Strain) Preparation and *In Vitro* Viability Assays

The *S. mansoni* life cycle was maintained at Swiss TPH, as previously described.^75^ Cercariae were obtained from infected *Biomphalaria glabrata* snails by exposing them to a strong light source for 3–4 h in pond water. Shed cercariae were mechanically transformed into NTS and then incubated at 37 °C with 5% CO_2_ in medium 199, supplemented with 5% FCS and 1% penicillin/streptomycin, for at least 12 h to a maximum of 24 h before use. Adult *S. mansoni* worms were collected by dissecting the mesenteric veins of infected NMRI mice on day 49 postinfection. The worms were then incubated in supplemented RPMI medium (5% FCS, 100 U/mL penicillin, and 100 μg/mL streptomycin) at 37 °C with 5% CO_2_ until they were needed.

Transparent flat-bottom 96- and 24-well plates (Sarstedt, Switzerland) were used for the NTS and adult worms, respectively. Compounds were initially tested at 20 and 10 μM in triplicate on NTS and repeated once; each well contained 30–40 NTS. Phenotypic reference points such as motility, morphology, and granularity were used to score incubated parasites’ overall viability (scores from 0 to 3).^75^ Parasites were observed via microscopic readout 72 h postincubation; compounds showing >50% activity at 10–20 μM were further tested at lower concentrations for IC_50_ determination (Calcusyn software version 2.0). Identified hits from the NTS screening were tested on *S. mansoni* adult worms. At least three worms (both sexes) were incubated with RPMI 1640 supplemented with 5% (v/v) FCS and 1% (v/v) penicillin/streptomycin at 37 °C with 5% CO_2_ for 72 h at concentrations of 20 and 10 μM. The experiment was conducted in duplicate and repeated; standard deviations were calculated from two wells. For all *in vitro* assays, negative controls (DMSO at the highest tested concentration) were included.

### Life Cycle of *S. mansoni* Maintenance, Viability Assays, and Egg Counts (Puerto Rican Strain)

A Puerto Rican strain of *S. mansoni*was maintained at the Schistodiscovery unit of CNR-IBBC in Monterotondo, Italy, by cycling within albino *Biomphalaria glabrata*, as the intermediate host and ICR (CD-1) outbred female mice as the definitive host, as previously described.^59^ Viability assays on adult worm pairs were based on a phenotyping scoring system as previously reported.^59,60^ Briefly, five adult pairs were incubated with selected compounds in 3 mL of DMEM supplemented with 10% fetal bovine serum. For each compound, three experiments were performed, and compounds were given to parasites only once without medium addition or replacement. DMSO (vehicle)-treated worms were used as control samples. Viability was monitored daily under a Leica Model MZ12 stereomicroscope for 3 days and viability scores (0–3) were assigned using the following criteria: score 3, for parasites showing plate-attached, good movements, clear aspect; score 2 for slower or diminished movements, darkening, minor tegumental damage; score 1 for heavily lowered movements, darkening, heavily damaged tegument; and score 0, for dead parasites with no movement. The total score for each sample was determined by the ratio of the sum of worm scores to the total number of worms examined. Worm couples’ unpairing was also recorded at 24, 48, and 72 h.

The eggs produced by worm pairs *in vitro* were counted on day 3 post-treatment using an inverted Leica DM IL microscope. Images were captured with a BX41 Olympus microscope and a brightfield objective 10× served by an Olympus DP23 microscope digital camera, visualized using “CellSens Entry” software. Egg maturation morphological score was assigned based on Vogel and Prata’s staging system of egg maturation as previously reported.^61,62^

### Confocal Microscopy Analysis

Carmine-Red staining and image analysis were performed as previously described.^63^ Images were acquired on a confocal laser scanning TCS SP5 microscope (Leica Microsystems, Wetzlar, Germany), using a 40× (NA = 1.25) oil-immersion lens with an optical pinhole at 1 AU. Argon laser at 488 nm was used as an excitation source, and fluorescence was recovered in the range of 500–700 nm. Images were collected as a single stack.

### Western Blot Analysis

Ten pairs of adult parasites were treated with selected Sirt inhibitors for either 24 or 48 h at partially lethal and sublethal concentrations. The concentrations of compounds were chosen based on the viability curve. Positive controls in the experiments included TSA, a pan-HDAC inhibitor, and salermide, a hSirt1/2 inhibitor, while DMSO (vehicle) served as negative control. Parasites were lysed in a 0.5% Triton PBS buffer by strokes, and the lysates were then centrifuged to collect the soluble fractions (cytosol). The insoluble fractions were extracted with 0.25 N HCl at +4 °C for 18 h, followed by neutralization with 1/10 volume of 2 N NaOH. Samples were analyzed using 15% SDS-PAGE and Western blotting. A ChemiDoc XRS Bio-Rad with a chemiluminescent camera and Bio-Rad ImageLab 4.0 software were utilized for the acquisition and analysis of images.

### Viability Assays on Mammalian Cells

The MTT viability assay was used to determine the viability of the mammalian cells BJ, human fibroblasts established from normal foreskin of a neonatal male from the American Type Culture Collection (ATCC; Manassas, Virginia, USA) (ATCC_CRL-2522), and L929 murine fibroblasts isolated from subcutaneous connective tissue (ATCC–CCL-1), as previously described.^64^ Cells were seeded into 96-well plates at densities of 10,000 (L929) and 20,000 (BJ) cells per well after an overnight incubation. They were then exposed to selected compounds at the indicated concentrations for 72 h. After exposure, the cells were gently washed and incubated with MTT, 3-(4,5-dimethyl-2-thiazolyl)-2,5-diphenyl-2*H*-tetrazolium bromide, for 4 h. The cells were then processed for color detection with DMSO. The resulting purple solution was spectrophotometrically measured at 570 nm using a Varioskan Lux instrument and Skanit software (Thermo Fisher Scientific). The optical density values for both assays were expressed as a percentage of cell survival and normalized with the value of cells treated with vehicle (DMSO). The data were analyzed using GraphPad Prism v9.5.1 software (San Diego, California, USA).

## Supplementary Material



## Notes

(Figure S1) Selected Sirt2 inhibitors that do not impair directly egg viability and maturation; (Figure S2) morphological alterations of *S. mansoni* (Puerto Rican strain) worm pairs treated with Sirt2 inhibitors ad day 6; (Figure S3) morphological alterations of *S. mansoni* (Puerto Rican strain) unpaired female worm; (Figure S4) selected Sirt2 inhibitors that do not impact histone H4 acetylation and total lysine acetylation; and (Table S1) elemental analyses for compounds **1**–**7** and **9** (PDF)

## Abbreviations

PZQ: praziquantel
NTS: newly transformed *Schistosomula*
