# Supplementary material for: Benzodeazaoxaflavin Sirtuin Inhibitors Inhibit Schistosoma mansoni Sirt2 and Cause Phenotypic Changes and Lethality in Schistosomula and Adult Worm Stages
Source: ACS Infect Dis. 2025 Oct 7;11(11):3115–27. doi: 10.1021/acsinfecdis.5c00515 (PMC12624727; doi:10.1021/acsinfecdis.5c00515)
Supplement: Supplementary file 1 [file id5c00515_si_001.pdf]

Supporting Information

Benzodeazaoxaflavin Sirtuin inhibitors inhibit *Schistosoma mansoni* Sirt2 and cause phenotypic changes and lethality in schistosomula and adult worm stages

By Roberto Gimmelli,[^¶] Giuliana Papoff, [^¶] Emanuele Fabbrizi,[†] Michela Guida, [†] Cristiana Lalli,[^] Fulvio Saccoccia, [^] Cécile Häberli[¥,+], Jennifer Keiser[¥,+], Daria Monaldi[#], Manfred Jung[#], Christophe Romier, [‖] Dante Rotili, [ϕ,Δ*] Antonello Mai, [†*] and Giovina Ruberti[^*]

^Institute of Biochemistry and Cell Biology, National Research Council (IBBC-CNR), Adriano Buzzati-Traverso Campus, Monterotondo (Rome) 00015, Italy.

†Department of Drug Chemistry and Technologies, Sapienza University of Rome, Rome 00185, Italy.

¥Swiss Tropical and Public Health Institute, Allschwil 4002,Switzerland.

+University of Basel, Basel 4001, Switzerland.

#Institute of Pharmaceutical Sciences, Albert-Ludwigs-Universität Freiburg, Freiburg 79104, Germany.

‖Départment de Biologie Structurale Intégrative, Université de Strasbourg, CNRS, INSERM, Institut de Génétique et de Biologie Moléculaire et Cellulaire (IGBMC), Illkirch Cedex 67404, France.

ϕDepartment of Science, Roma Tre University, Viale Guglielmo Marconi 446, Rome 00146, Italy.

ΔBiostructures and Biosystems National Institute (INBB), Via dei Carpegna 19, Rome 00165, Italy.

¶ These authors equally contributed to this work

* Corresponding authors

Email: dante.rotili@uniroma3.it

Email: antonello.mai@uniroma1.it

Email: giovina.ruberti@cnr.it

Table of Content:

Figure S1. Selected Sirt2 inhibitors do not impair directly egg viability and maturation.

Figure S2. Morphological alterations of *S. mansoni* (Puerto Rican strain) worm pairs treated with Sirt2 inhibitors ad day 6.

Figure S3. Morphological alterations of *S. mansoni* (Puerto Rican strain) on unpaired female worm.

Figure S4. Selected Sirt2 inhibitors do not impact histone H4 acetylation and total lysine-acetylation.

Table S1. Elemental analyses for compounds **1-7**, **9**.

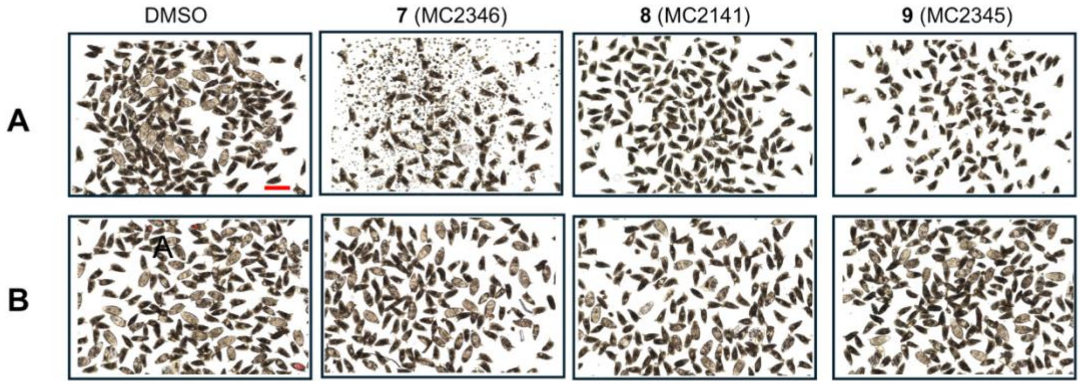

Figure S1. Selected Sirt2 inhibitors do not impair directly egg viability and maturation. (A) Eggs of treated worm pairs; (B) IVLEs were collected and exposed to vehicle (DMSO) at the same volume as samples treated with Sirt2 inhibitors at the concentration of 10 µM. Representative pictures of IVLEs treated for 72 hrs are shown. Scale bar = 200 µm.

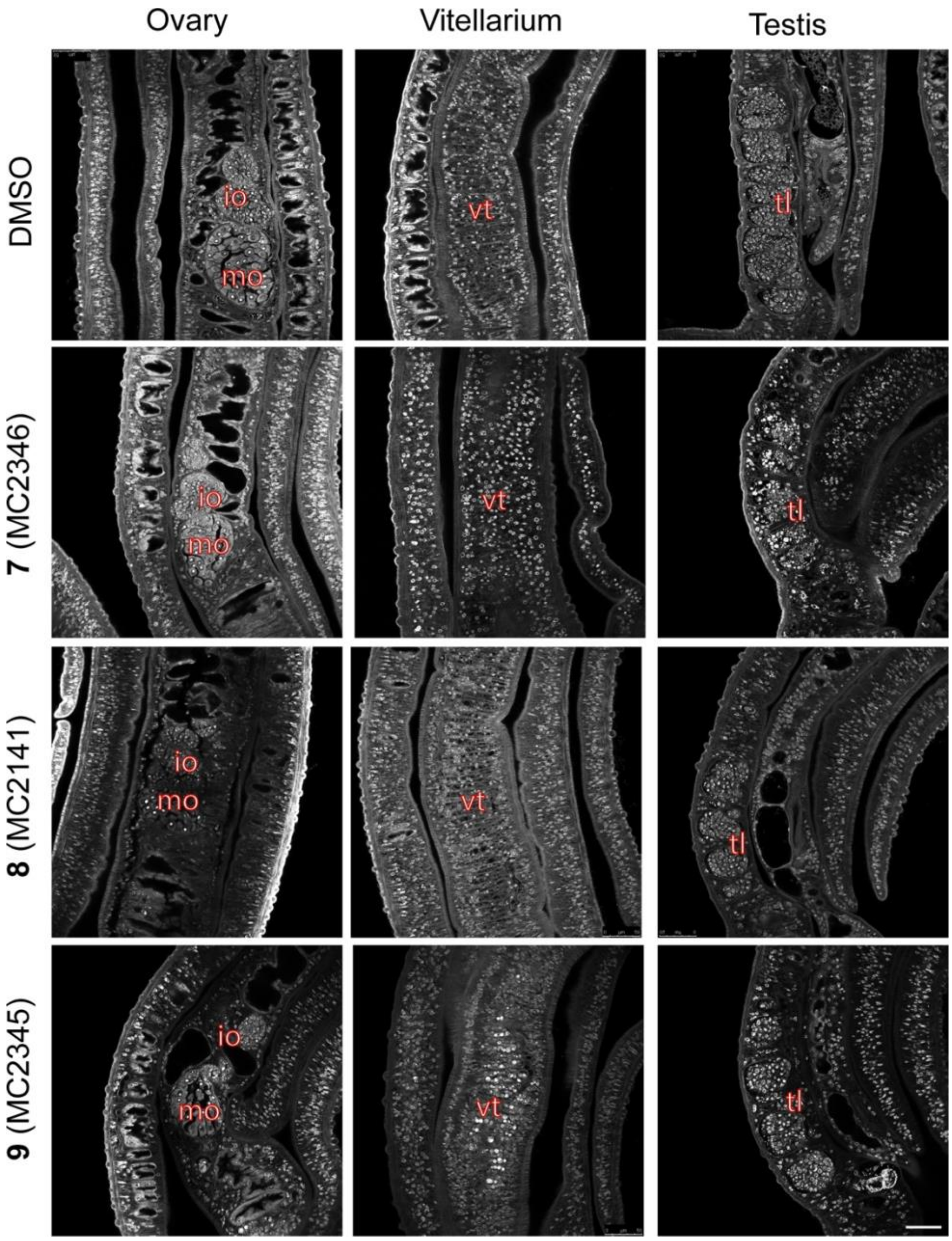

Figure S2. Morphological alterations of *S. mansoni* (Puerto Rican strain) worm pairs treated with Sirt2 inhibitors ad day 6. The images are representative of 3–4 worm pairs treated with DMSO, and the indicated inhibitors at the concentration of 10 µM with the Sirt2 inhibitors indicated, for 6 days. The ovary, vitellarium and testis are shown. Immature oocytes (io), mature oocytes (mo), ootype (ot), and testicular lobes (tl) are labeled. Scale bars 50 µm.

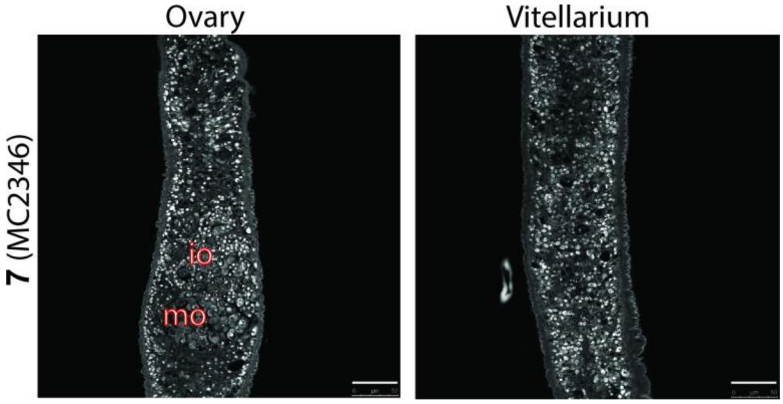

Figure S3. Morphological alterations of *S. mansoni* (Puerto Rican strain) unpaired female worm. The images are representative of unpaired females obtained after treatment of worm pairs with compound **7** (MC2346) (20 µM) for 6 days. The ovary and vitellarium are shown, and immature oocytes (io) and mature oocytes (mo) are labelled. Scale bars: 50 µm.

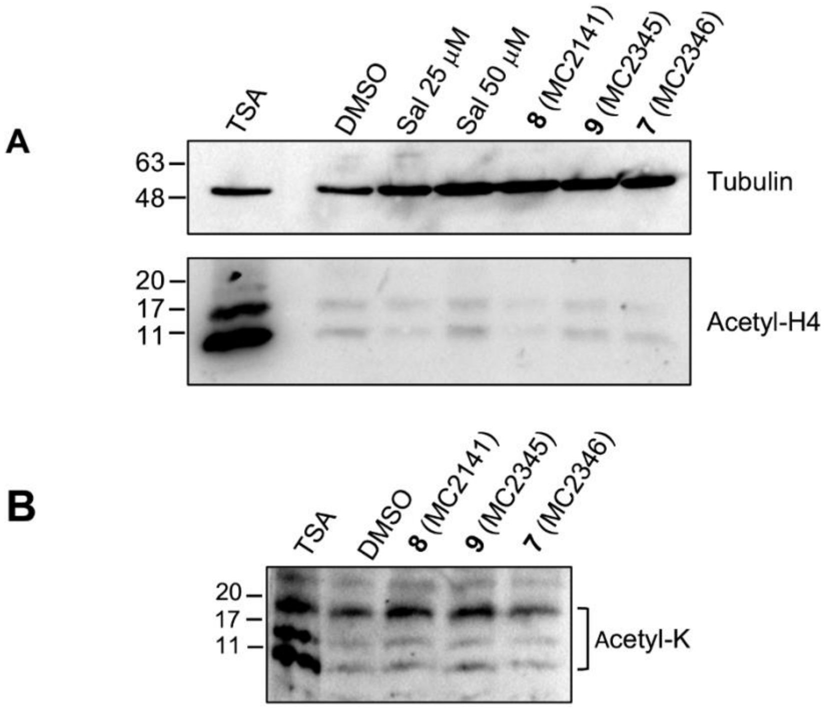

Figure S4. Selected Sirt2 inhibitors do not impact histone H4 acetylation and total lysine-acetylation. A) Representative immunoblots of histone-enriched protein fractions from adult worm pairs of *S. mansoni* (Puerto Rican strain) treated with 10 µM of the selected compounds for 48 hrs. Controls included DMSO (vehicle), TSA (1 µM, 24 hrs) and Salermide (Sal 25 and 50 µM, 48 hrs) were used as controls. Tubulin signal served as loading control. B) Total levels of Lysine (K)-acetylation are shown.

Table S1. Elemental analyses for compounds 1-7, 9.

compd	MW	% calculated			% found		
		C	H	N	C	H	N
**1**	264.24	68.18	3.05	10.60	68.32	3.12	10.41
**2**	278.27	69.06	3.62	10.07	68.88	3.57	10.34
**3**	292.29	69.86	4.14	9.58	69.59	4.01	9.85
**4**	304.31	71.05	3.97	9.21	71.29	4.04	8.97
**5**	306.32	70.58	4.61	9.15	70.77	4.72	8.92
**6**	320.35	71.24	5.03	8.74	71.55	5.10	8.49
**7**	346.39	72.82	5.24	8.09	72.54	5.11	8.27
**9**	354.37	74.57	3.98	7.91	74.69	4.04	7.77
